# Medicinal importance of grapefruit juice and its interaction with various drugs

**DOI:** 10.1186/1475-2891-6-33

**Published:** 2007-10-30

**Authors:** Jawad Kiani, Sardar Z Imam

**Affiliations:** 1 Medical College, Aga Khan University, Stadium Road, Karachi, Pakistan

## Abstract

Grapefruit juice is consumed widely in today's health conscious world as a protector against cardiovascular diseases and cancers. It has however, been found to be an inhibitor of the intestinal cytochrome P – 450 3A4 system, which is responsible for the first pass metabolism of many drugs. The P – glycoprotein pump, found in the brush border of the intestinal wall which transports many of these cytochrome P – 450 3A4 substrates, has also been implicated to be inhibited by grapefruit juice. By inhibiting these enzyme systems, grapefruit juice alters the pharmacokinetics of a variety of medications, leading to elevation of their serum concentrations. Most notable are its effects on the calcium channel antagonist and the statin group of drugs. In the case of many drugs, the increased serum concentration has been found to be associated with increased frequency of dose dependent adverse effects. In this review, we have discussed the phytochemistry of grapefruit juice, the various drugs involved in the drug – grapefruit juice eraction with their mechanisms of action and have presented the clinical implications of these interactions.

## Introduction

The grapefruit, thought to be a cross between an orange and a shaddock, was developed in the West Indies in the early 1700s and first introduced to Florida in the 1820s. Since the early part of the 20th century, mutant strains of white grapefruit have appeared with pink to slightly reddish colour, and have been propagated by citriculturists into several strains of grapefruit. The three major types of grapefruit that exist today are white, pink/red and ruby/rio red varieties. Grapefruit juice combines the sweet and tangy flavour of the orange and shaddock and also provides up to 69% of the RDA for vitamin C along with as many as 250 mg of Potassium [1].

However, the wide consumption of grapefruit juice cannot entirely be attributed to its taste, and nutritive value. In fact, much of the enthusiasm in its use stems from medical research that has suggested that grapefruit juice reduces atherosclerotic plaque formation [2] and inhibits breast cancer cell proliferation and mammary cell tumorigenesis [3,4]. Traditionally grapefruit juice has been found to contain antioxidant, antinitrosaminic, antiseptic, aperitif, cardiotonic, detoxicant, hypocholesterolemic, sedative and stomachic activities. In the light of its above activities, it has been traditionally indicated throughout time for anorexia, bacteria, benign prostatic hypertrophy, cancers (breast, colon, prostate, lung, skin and throat), candida, cold, diabetes, dysuria, high cholesterol, infection, insomnia, mycobacterium, mycosis, nervousness, pseudomonas, rheumatism, staphylococcus and yeast.

However, as many as fifteen years ago, investigators found that grapefruit juice can markedly augment oral drug bioavailability. This was an unexpected observation from an interaction study between the dihydropyridine calcium channel antagonist, felodipine, and ethanol in which grapefruit juice was used as a flavour supplement to mask the taste of the ethanol [5]. Studies that followed, confirmed that grapefruit juice significantly increased the oral bioavailability of felodipine [6,7]. Subsequent studies probed the constituents of grapefruit juice, its interaction with various other drugs and the mechanisms of action of those interactions. Several grapefruit juice-drug interactions were discovered and these remain a potential concern especially since the juice and drugs are often consumed together at breakfast. An increasing number of adverse drug reactions might be avoided on the basis of knowledge about the interaction of grapefruit juice and relevant drugs. Therefore, patients need to be educated about the hazards (and advantages) of grapefruit interaction with medication. In recent years, more drugs have been investigated for their interaction with grapefruit juice and new models have been proposed for the mechanism of such interaction. This article presents a simplistic summary of most examples of such interactions and also explores the phytochemistry and possible mechanisms of action involved in drug-grapefruit juice interactions in light of recent studies on this subject.

## Mechanism of action

The mechanism of action of this interaction involves inhibition of the CYP 3A4, a member of the cytochrome P 450 (CYP) enzyme system. CYP is a large multigene family of heme-containing enzymes located in the endoplasmic reticulum of cells throughout the body. It is especially concentrated in the liver and intestinal wall where it is involved in oxidative biotransformation of various endogenous and exogenous substances. CYP 3A isoforms constitute 70% of CYP enzymes in enterocytes [8,9]. P-glycoprotein (Pgp), a member of the ABC (adenosine triphosphate-binding cassette), is another membrane transporter located in the apical brush border of enterocytes. Once taken up by the enterocytes, a lipophilic drug may be metabolized by CYP 3A4 or be pumped back into the lumen by the Pgp. Hence the oral delivery of many drugs is limited by the actions of CYP 3 A4 or Pgp. Metabolism by the CYP 3A4 will also occur in the liver before the drug finally enters the systemic circulation. Grapefruit juice causes inhibition of CYP 3A4 and thus serves to increase the bioavalability of the drug by decreasing its pre-systemic metabolism [10]. This action is in essence, similar to that caused by CYP-inhibiting drugs like itraconazole, ketoconazole and erythromycin [11-13].

Grapefruit juice causes quick and irreversible sustained inhibition of the CYP system, possibly by greatly accelerating the degradation of these enzymes while also reducing translation from its mRNA. However, the process of transcription of mRNA from the cell DNA is not affected. Overall, grapefruit juice reduces the levels of CYP 3A4 in the cells by as much as 47% within four hours of ingestion of grapefruit juice with the resultant increased bioavailability being maintained for as long as 24 hours, by which time 30% of its effect is still detectable [14-17]. It has been observed that decreased content of CYP3A4 was not associated with increased CYP3A4 mRNA, probably indicating the absence of a feedback mechanism for CYP3A4 expression. Restoration of CYP3A4 activity would therefore require denovo synthesis or enterocyte replacement, accounting for the prolonged duration of the actions of grapefruit juice [18].

Grapefruit juice shows a high variability of the magnitude of effect among individuals. This variability is dependent upon inherent differences in enteric CYP3A4 protein expression such that individuals with highest baseline CYP3A4 have the highest proportional increase [19,20]. However, the effects of grapefruit juice are predominantly on the intestinal CYP rather than hepatic CYP. This is shown by the fact that most of the drugs that are involved in interaction with grapefruit juice undergo their primary metabolism at the intestinal level and in usual quantity, grapefruit juice does not affect the pharmacokinetics of these drugs when they are administered intravenously. Furthermore, while it increases the area under the plasma concentration-time curve (AUC), it has no significant effect on the half life of the drugs [10,21-23].

In contrast to the clear inhibitory effects of grapefruit juice on CYP 3A4, the effects of grapefruit juice on Pgp are controversial, ranging from activation to inhibition. Earlier results have shown grapefruit juice to cause activation of Pgp in vitro [24]. Any such activation in vivo will mean a greater efflux of the drug back into the lumen, thereby decreasing the oral bioavailability of that drug and at least partially, if not completely offsetting the effects produced by the inhibition of CYP system of enzymes. This is taken as an explanation for the less-than-expected increase in the bioavailability of drugs that are established substrates of Pgp [24]. However, grapefruit juice does not change the absorption of digoxin, a prototypical P-glycoprotein substrate, likely because it has high inherent oral bioavailability [17,25]. However, recent studies have demonstrated the inhibition of Pgp by grapefruit juice both by its down-regulation and inhibition of function [26,27]. For example, grapefruit juice increases the bioavailability of cyclosporine. This effect is thought to be primarily though Pgp inhibition (instead of CYP3A4 inhibition) since orange juice mediated reduction in enterocyte CYP3A4 concentrations did not produce a similar increase in bioavailability [17]. In fact, grapefruit juice has also shown inhibition of multidrug resistant protein 2 (MRP2), an efflux protein closely related to Pgp in terms of its expression and function [26].

Yet, in spite of all what is known, the mechanism of action of grapefruit juice-drug interaction requires further investigation. Investigators still need to determine for certainty any in vivo effect of grapefruit juice on Pgp. One study [28] has also reported the action of grapefruit juice independent of its actions on Pgp and CYP 3A4. This also requires further investigation. Similarly, grapefruit and even orange juice have also recently been shown to be potent in vitro inhibitors of a number of organic anion-transporting polypeptides (OATPs) that are involved in apical-to-basal transport of drugs in the small intestine [17,18,25,29]. They were also found to decrease the absorption of the non-metabolized OATP substrate, fexofenadine hence pointing towards inhibition of intestinal uptake transporters by fruit juices to decrease drug bioavailability. This newly proposed mechanism of action and its effect vis a vis various medications also demands further investigation [25,29].

Assessment of the in vitro CYP inhibition potential for these natural products has important implications for predicting the likelihood of natural product-drug interactions if these products are taken concomitantly. The susceptibility of CYP3A4 to modulation by food constituents may be related to its high level of expression in the intestine, as well as its broad substrate specificity. Reported ethnic differences in the activity of this enzyme may be partly due to dietary factors. Food-drug interactions involving CYP1A2, CYP2E1, glucuronosyltransferases and glutathione S-transferases have also been documented, although most of these interactions are modest in magnitude and clinically relevant only for drugs that have a narrow therapeutic range. Recently, interactions involving drug transporters, including P-glycoprotein and the organic anion transporting polypeptide, have also been identified. Hence a lot of food varieties have the potential to require dosage adjustment to maintain drug concentrations within their therapeutic windows, especially with drugs that have a high first pass degradation [30]. Further research is needed to determine the scope, magnitude and clinical importance of food effects on drug metabolism and transport.

## Relevant phytochemistry

Another area in which the search for definite answers continues, is the quest to find the active constituents of grapefruit juice that are responsible for its actions on CYP enzyme systems and Pgp. The components of grapefruit juice that are responsible for clinical drug interactions have yet to be fully determined but the compounds thought to be responsible for this action include flavonoid glycosides (narirutin, naringin, naringinen, quercetin, kaemferol, hesperidin, neohesperidin, didymin, and poncirin) [8,31-34], furanocoumarins (6',7'-dihydroxybergamottin, bergamottin) and sesquiterpen (nootkatone)[8,22,32,35,36].

Flavanoids exist in grapefruit juice in the form of glycosides, with naringin being the most abundant. Upon ingestion, these are converted to aglycones and sugars by the action of intestinal flora. Being polyphenolic and electron rich, these compounds can theoretically inhibit the CYP enzymes. However, studies have at most shown an in vitro effect by these compounds on the these enzymes and have failed to identify any in vivo effect by them [37,38], leading to an implication that they are probably not the main active ingredients of grapefruit juice [1,39]. Studies have even failed to demonstrate any sort of activity in naringin although its metabolite naringinin was observed to be active in vitro. Yet, because of their huge quantities in grapefruit juice, and the fact that naringin is not present in other citrus juices, flavanoids remain a subject of research.

The main focus at present, however, is on furanocoumarins. This group includes Bergamottin, its derivative 6' 7' dihydroxybergamottin (DHB) and a host of other compounds [40]. Controversy still exists on the degree of their role in the inhibitory effects of grapefruit juice. Several studies have shown DHB [23,35,40] and to an extent Bergamottin [23] to be important contributors to the grapefruit juice effect. In one study, the inhibitory potency of DHB and four recently isolated furanocoumarins, when mixed with one another, almost approached that of grapefruit juice. Omission of any of the components resulted in decreased potency, suggesting that all major furanocoumarins contribute to the inhibitory effects of grapefruit juice [40]. However, others have suggested that DHB and Bergamottin are not the primary substances responsible for inhibition of CYP activity clinically [41,42]. For now, this topic also remains a subject of intense research.

## Drug-grapefruit juice interactions

### Anti-hypertensive drugs and amiodarone

1,4-Dihydropyridine calcium antagonists are lipid soluble drugs used in the treatment of essential hypertension and angina pectoris and metabolized in vivo by CYP3A4. Since the effects of grapefruit juice were first noticed with felodipine, this class of drugs has been intensively studied with grapefruit juice. The degree to which the intestinal CYP system metabolizes this class of drugs and affects their oral bioavailability varies markedly. In a study done by Lundahl J et al., it was found that the intake of grapefruit juice led to an increase in the oral bioavailability by 112% [43]. However, this study also found out that the intravenous pharmacokinetics of felodipine were not significantly altered with grapefruit juice. The main acute effect of the grapefruit juice on the plasma concentrations of felodipine was believed to be mediated by inhibition of gut wall metabolism. Grapefruit juice-felodipine interaction increases with increasing frequency and amount of grapefruit juice ingestion, hence it has been determined that an interval of 2–3 days between grapefruit juice intake and felodipine administration is necessary if the interaction is to be avoided [44]. Blood pressure responses to felodipine with grapefruit juice have also been assessed in the elderly and the systolic and diastolic blood pressures were found to be lower with grapefruit juice in the single-dose state, whereas they were not different between treatments in the steady-state dose [45]. The different blood pressure results between the studies can be explained by felodipine concentration-blood pressure response relationships. The elderly should be particularly cautioned about concomitant grapefruit juice and felodipine ingestion.

In the benzothiazepine calcium channel antagonists group, diltiazem has been found to have an increased bioavailability on co administration of a single intake of grapefruit juice. Inhibition of intestinal metabolism and/or P-glycoprotein efflux transport was believed to be possibly responsible for this effect [46]. However in contrast to this, another study showed the bioavailability to be unchanged with grapefruit juice suggesting that factors other than biotransformation may be contributing [47].

Compared with water, grapefruit juice increased the maximum concentration of nisoldipine and reduced the time to reach maximum nisoldipine concentration [48]. However, the effects of grapefruit pulp intake were smaller than those produced by grapefruit juice intake, indicating that grapefruit pulp and juice have different effects on the pharmacokinetics [49].

A clinical study was performed to see the duration of this interaction in the body. Eight healthy volunteers were given grapefruit juice at 14, 38, 72 and 96 hours. Compared with the control group, the maximum plasma concentration of nisoldipine was significantly increased after grapefruit juice intake in at 0 and 14 hours, and the plasma concentration was significantly increased at each time till 72 hours [50]. It is therefore necessary to withhold grapefruit juice for at least 3 days before administration of the drug to prevent grapefruit juice -nisoldipine interaction.

Regarding verapamil, there are conflicting reports about its interaction with grapefruit juice. One study showed an increase in its bioavailability at steady state [51] while another showed no significant change in pharmacokinetics on a single administration.

ACE-inhibitors like enalapril, captopril, lisinopril and ramipril have not shown any interaction with grapefruit juice although such an interaction might be possible with angiotensin II type 1 receptor antagonists like losartan and valsartan [18]. Thiazide diuretics and alph 1 adrenergic antagonists (doxazosin, terazosin, prazosin) have also shown no interaction with grapefruit juice [18].

Amiodarone, an antiarrythmic, is metabolized by CYP3A to N-desethylamiodarone (N-DEA), a metabolite more potent than the parent drug [17]. On interaction with grapefruit juice, there has been shown to be complete inhibition of N-DEA production [52] leading to an overall decrease in the arrythmogenic side effects of amiodarone [17]. These results are in agreement with in vitro data pointing to the involvement of CYP3A in the metabolism of amiodarone and other Ca antagonists, suggesting that this interaction should be taken into account when prescribing this antiarrhythmic drug. Similarly grapefruit juice has been found to increase oral nimodipine bioavailability [53]. The same cannot be said of amlodipine, on which grapefruit juice has no appreciable effect [54].

One of the possible active ingredients in commercial grapefruit juice is Bergamottin, as mentioned before. This was determined after studying the effects of the furanocoumarin derivative on nifedipine (NFP) pharmacokinetics, suggesting that bergamottin in grapefruit might be the substance that elevates the NFP plasma concentrations [55].

Further studies have also been done to determine if even unprocessed grapefruit could cause drug interactions. It has been shown that unprocessed grapefruit can cause a drug interaction with felodipine [56]. 6', 7'-Dihydroxybergamottin and naringin were implicated to be more important in this case because they are present in higher concentrations in grapefruit extracts.

### Antimicrobials

With antivirals, authors concluded that concomitant administration of grapefruit juice increases gastric pH and delays indinavir absorption but does not uniformly affect the systemic bioavailability of indinavir in HIV-infected subjects [57,58]. Similarly grapefruit juice has been shown clinically to not significantly affect amprenavir pharmacokinetics [59]. It is suggested that this may be because the primary metabolism of these drugs is not in the small intestine.

On the other hand regarding saquinavir, it has been shown that grapefruit juice increases the bioavailability of saquinavir without affecting its clearance, suggesting that inhibition of intestinal CYP3A4 may contribute [60]. And since the antiretroviral effect of saquinavir is dose-dependent, it has been suggested that inhibition of CYP3A4 may represent a way to enhance its effectiveness without increasing the dose.

Amongst anti malarials, grapefruit juice significantly increases the oral bioavailability of artemether but does not prevent the time-dependent reduction in bioavailability or elimination half-life, suggesting a role for intestinal CYP3A4 in the presystemic metabolism of artemether [61,62]. Similar results have also been seen after a single oral dose of praziquantel with 250 ml of grapefruit juice [63].

Quinine appears to be unaffected in its pharmacokinetics. Since quinine is a low clearance drug with a relatively high oral bioavailability, and is primarily metabolised by human liver CYP3A4, the lack of effect of grapefruit juice on quinine pharmacokinetics again supports the view that the site of CYP inhibition by grapefruit juice is mainly in the gut [17,64]. However for quinidine, grapefruit juice reduces its total clearance and increases the elimination half-life by 19% [65].

In antibiotics, administration of grapefruit juice increased the time to peak concentration of clarithromycin but did not affect other pharmacokinetic parameters [66] while in antiparasitics, albendazole showed an increase in bioavailability upon administration of grapefruit juice [67].

### Benzodiazepines and CNS drugs

A marked interaction between oral midazolam and grapefruit juice has been found and the data is consistent again with a reduced first-pass metabolism of midazolam, resulting in increased bioavailability of midazolam [68,69]. The clinical importance of this is especially for patients with other causes for increased midazolam bioavailability such as advanced age, cirrhosis of the liver, and administration of other inhibitors of cytochrome P450. Thus, patients with liver cirrhosis are more dependent on the intestine for metabolism of CYP3A4 substrates than subjects with normal liver function. Another important implication of this interaction is in dentistry. Oral midazolam is a frequently used sedative in pediatric dentistry. Although an oral form of midazolam is now commercially available, some practitioners continue to use the IV midazolam as an oral medication. If the injectible form of midazolam is administered orally, its bitter taste requires the use of a flavoring agent like grapefruit juice. This results in increased blood plasma levels of midazolam causing excessive levels of sedation for the pediatric patient. Grapefruit juice therefore should be contraindicated for use with oral midazolam especially in such patients [70]. Similar results have also been seen with triazolam [71].

One study however, did show that grapefruit juice did not have any particular interaction with oral doses of 10 mg midazolam and 0.25 mg triazolam in healthy young subjects [72]. However, since more studies have determined increases in midazolam and triazolam bioavailbility, grapefruit juice should be administered with caution with these drugs. However, alprazolam remains unaffected in pharmacokinetics or pharmacodynamics due to its high bioavailability [73].

Among antipsychotics, clozapine remained unaffected after consumption of regular-strength grapefruit juice, usually taken as 250 mL b.i.d., for 14 days [74]. One reason for this is that enzymes other than CYP3A4 also mediate clozapine disposition. Haloperidol remains unaffected by grapefruit juice [75].

In anti convulsants, grapefruit juice increases the bioavailability of carbamazepine [76] but does not affect the pharmacokinetics of phenytoin [77].

Grapefruit juice considerably increases plasma buspirone concentrations [78] and also increases sertraline bioavailability [79]. Grapefruit juice therefore should be contraindicated during administration of buspirone and sertraline.

### Antihistamines and Serotonin Analogs

A number of studies have shown that a single glass of grapefruit juice produced an individual-dependent, variable increase in the systemic bioavailability of cisapride by inhibition of intestinal cytochrome P450 3A4 (CYP3A4) activity. [80-82] It has therefore been recommended that concomitant use of high amounts of grapefruit juice with cisapride should be avoided, at least in patients with risk factors for cardiac arrhythmia.

The effect of grapefruit juice on racemic nitrendipine was also to increase its bioavailability and it was found that it inhibits the stereoselective metabolism of nitrendipine in humans [83].

Regarding terfenidine, the ingestion of grapefruit juice leads to its enhanced systemic bioavailability [84,85]. This is especially important because the raised levels of terfenidine can prolong the QT interval in the electrocardiogram sufficiently to precipitate the ventricular arrhythmia of Torsade-des-pointes [86]. Incidentally, both terfenidine and cisapride have been globally withdrawn from the market due to serious cardiac arrythmias precipitated by their interaction with other drugs if simultaneously taken.

### Statins and other cholesterol-lowering agents

Taking simvastatin first, the active ingredient *bergamottin *has been shown to inhibit simvastatin (SV) metabolism and increase the serum concentrations of simvastatin and its active metabolite simvastatin acid, and, to a lesser extent, those of active and total HMG-CoA reductase inhibitors [87,88]. The probable mechanism of this interaction was also the inhibition of CYP3A4-mediated first-pass metabolism of simvastatin by grapefruit juice in the small intestine. Bergamottin (BG) and naringenin (NRG) could therefore be applied as markers in food-drug interaction studies in order to adjust posology and the dose of simvastatin should be accordingly reduced.

Another study further found out that, when simvastatin is taken 24 hours after ingestion of "high-dose" grapefruit juice, the effect on the concentration of simvastatin is only about 10% of the effect observed during concomitant intake of grapefruit juice and simvastatin. It was also shown that the interaction potential of even high amounts of grapefruit juice with CYP3A4 substrates dissipates within 3 to 7 days after ingestion of the last dose of grapefruit juice [89].

The grapefruit juice effect has also been studied on lovastatin. Lovastatin and its active metabolite, lovastatin acid had greatly increased serum concentrations after grapefruit juice administration [90]. However, one other study has shown a minimal effect of a glass of regular-strength grapefruit juice on plasma concentration after a 40 mg evening dose of lovastatin [91].

Although grapefruit juice also increases the AUC of atorvastatin, the actual increase in activity is fairly modest, possibly due to a simultaneous effect of decreasing the AUC of active metabolites of atorvastatin [17]. Regardless, grapefruit juice should not be concomitantly ingested with atorvastatin, lovastatin or simvastatin. On the other hand, pravastatin, fluvastatin and rosuvastatin are three statin drugs that have been shown not to interact with grapefruit juice [18]. These may be useful alternatives in settings where there is a concern regarding potential interaction with grapefruit juice.

Other cholesterol-lowering agents like nicotinic acid and common fibric acid derivatives and bile acid sequestrants have shown no interaction, and therefore may be safely used, with grapefruit juice [18].

### Chemotherapeutics

In patients with autoimmune diseases, the effect of chronic grapefruit juice administration on steady state blood concentrations of cyclosporine and metabolites is an increase in both parent and metabolite profiles [92]. This interaction was studied in renal transplant recipients. Administration of cyclosporine with grapefruit juice compared with water induced a moderate, butsignificant increase in the systemic exposure of cyclosporine [93,94]. Most of these studies involving cyclosporine were done on adult patients. However, one study was also done in the paediatric population. This study showed that alterations in cyclosporine absorption and elimination only occur with concurrent grapefruit juice ingestion when stable pediatric renal transplant patients are taking the oral cyclosporine solution, but not the microemulsion formulation [95].

Regarding prednisolone and etoposide, grapefruit juice has been found to have no significant effect on the metabolism of prednisolone [96] but in the case of etoposide, it has been shown to decrease its bioavailability [97].

## Conclusion

In light of the wide ranging effects of grapefruit juice on the pharmacokinetics of various drugs, physicians need to be aware of these interactions and should make an attempt to warn and educate their patients regarding potential consequences of concomitant ingestion of these two items. Patient-to-patient variability should be kept in mind and elderly should be particularly warned about these interactions since they are more prone to grapefruit juice-drug interactions [17]. Physicians should also consider using these effects to their own advantage in order to reduce the dosage requirements of certain drugs. However, since further research is required into the mechanism of action of grapefruit juice, it is still premature to recommend it as an adjunctive booster with other drugs.

## Abbreviations

AUC = area under the plasma concentration-time curve 

CYP = cytochrome P -450

DEA = desethylamiodarone

DHB = dihydroxybergamottin

HMG – CoA = 3 – hydroxyl – 3 – methylglutaryl coenzyme A

NFP = nifedipine

OATP = organic anion-transporting polypeptides

Pgp = P – glycoprotein

RDA = Recommended daily allowance

SV = simvastatin

## Competing interests

The author(s) declare that they have no competing interests.

## Authors' contributions

Both authors contributed equally.
